# Society Meetings

**Published:** 1916-03

**Authors:** 


### SOCIETY MEETINGS

Brief reports and announcements of meetings of societies of Western New York, and those of general scope, are requested from Secretaries. Copy should be on hand the fifteenth of the month. Full transactions will be published at cost of composition.

---

**DOCTOR: Is your Society properly represented here? If not, please bring our standing announcement to the attention of the members.**

---

**Presidents, Secretaries and Treasurers of County Societies.**

Allegany, C. W. O'Donnell, Andover; C. R. Bowen, Almond; C. R. Bowen, Almond.

Brooms, H. C. Sears, Binghamton; H. DeW. Watson, Binghamton; F. M. Dyer, Binghamton.

Cattaraugus, M. E. Fisher, Delevan; H. H. Ashley, Machias; H. H. Ashley, Machias.

Cayuga, J. M. Jenkins, Auburn; G. H. Beers, Auburn; F. A. Lewis, Auburn.

Chautauqua, A. W. Dods, Fredonia; J. W. Morris, Jamestown; G. F. Smith, Falconer.

Chemung, R. H. V. Dann, Elmira; F. M. Flood, Elmira; H. W. Fudge, Elmira.

Chenango, A. R. Morse, Oxford; P. B. Brooks, Norwich; J. B. Drake, Norwich.

Erie, F. W. Barrows, Buffalo; F. C. Gram, Buffalo; A. T. Lytle, Buffalo.

Genesee, E. F. Hagedorn, Gloversville; H. H. Oaksford, Gloversville; J. D. Vedder, Johnstown.

Herkimer, J. W. Graves, Herkimer; A. W. Suiter, Herkimer; G. W. Graves, Herkimer.

Jefferson, J. D. Olin, Watertown; C. E. Pierce, Watertown; P. E. Thornhill, Watertown.

Livingston, H. B. Marvin, Lima; G. K. Collier, Sonyea; G. K. Collier, Sonyea.

Madison, * * * * * * * ;G. W. Miles, Oneida; N. O. Brooks, Oneida.

Monroe, F. W. Seymour, Rochester; C. W. Hennington, Rochester; C. C. Sutter, Rochester.

Niagara, H. A. Wilmot, Middleport; L. R. Hurlbut, Lockport; W. A. Scott, Niagara Falls.

Oneida, W. B. Roemer, Utica; D. E. Pugh, Utica; T. W. Clarke, Utica.

Onondaga, G. M. Price, Syracuse; E. V. Sweet, Syracuse; R. J. Stoup, Syracuse.

Ontario, B. T. McDowell, Bristol Center; D. A. Eiseline, Shortsville; D. A. Eiseline, Shortsville.

Orleans, J. Dugan, Albion; G. F. Rogan, Medina; G. F. Rogan, Medina.

Seneca, A. Letellier, Seneca Falls; F. W. Lester, Seneca Falls; F. W. Lester, Seneca Falls.

Steuben, L. M. Kysor, Hornell; B. R. Wakeman, Hornell; B. R. Wakeman, Hornell.

Sullivan, A. B. Sullivan, Liberty; C. S. Payne, Liberty; C. S. Payne, Liberty.

Tompkins, W. G. Fish, Ithaca; C. F. Denman, Ithaca; J. W. Judd, Ithaca.

Wayne, E. A. Nevin, Newark; R. Sheldon, Lyons; R. Sheldon, Lyons.

Wyoming, J. R. Brownell, Perry; J. F. Crawford, Warsaw; J. F. Crawford, Warsaw.

Yates, G. H. Leader, Penn Yan; G. E. Walker, Dresden; E. M. Scherer, Penn Yan.

---

### Medical Association of Central New York.

Minutes of 47th Annual Meeting continued from last issue.

The PRESIDENT: Dr. Rochester.

DR. ROCHESTER: Mr. Chairman, I came in so late that I am really not able to discuss the paper.

The PRESIDENT: Dr. Coon, will you have something to say on the X-Ray?

DR. COON: I do not want to let the opportunity go to congratulate Dr. Palmer upon the X-Rays he has shown, and also upon the question of diagnosis. There is so much that can be done by the X-Ray man. Those of us who have had experience in making lantern slides from X-Ray negatives appreciate the difficulty in bringing out the prime features, the prime detail which we wish to show, and on which so much depends for a diagnosis. By the best processes it cannot all be placed on the slides, and for that reason I want to congratulate Dr. Palmer upon the excellence of the slides as shown here. A great many of them were very, very clear. The question of the diagnosis of the various lesions of the metastyle, the heart as well as the stomach and digestive tract, is yet an open one in a great many respects, a great many things have not been decided, but all of us must appreciate the value of the X-Ray in the study of practically all of these cases. A tuberculous lesion of the lung can be shown very early; some even claim it can be shown before the ordinary expert diagnostician can do it by percussion, palpation and other means of study. A lot can be done and is done with the study of heart lesions. Of course while it probably will never be possible to show the action of the heart valves, yet any serious abnormality of that heart can be shown. Certainly this is true of the stomach and the organs below the diaphragm, a lot can be shown, and I want to thank Dr. Palmer for the excellent exposition he has given.

DR. ALLEN JONES, Rochester: Mr. President, these papers by the doctors from Toronto, and Dr. Palmer's paper, are, as has been said, very valuable. I am reminded of the vast changes which have taken place in clinical medicine in the last few years. A good many of us here remember well when we were dependent for diagnosis upon our physical methods and upon symptomotology, and while those are very valuable today, diagnosis has been defined and made very much more accurate and clear by the methods which have been presented today. Without symptomotology and without physical diagnosis, the electro-cardiograph and the polygraph and the Roentgen Ray amplified, enlarged and certified tremendously, they have made possible absolute and distinct advances in medicine, comparable with the advances which have been made in surgery. These methods are so extremely valuable that they should be at the command of all of those who are practicing medicine, and the time has come when I am sure we will all not only desire to command these methods, but will make it possible for us to command them. That is all that I wish to say, Mr. President.

The PRESIDENT: Dr. Doust, do you wish to add anything to the X-Ray side of this discussion?

DR. DOUST, Syracuse: Mr. President, I am sure that these pictures, particularly of the lung, have been very instructive to all of us. There is one thing I think that we should remember, and which Dr. Palmer spoke of, and which may be emphasized, and that is the fact that in the diagnosis of pulmonary tuberculosis by the X-Ray, particularly the incipient conditions, we must remember that the X-Ray does not differentiate between an active process and a latent process. Of course, we all of us know that a great number of individuals, adults, have latent pulmonary tuberculosis, have latent lesions, and if we depend too much upon the X-Ray in the differentiation between an active and a latent condition we will many times be led astray.

The PRESIDENT: Is there any general discussion? I have been calling names of leaders, but not with the idea of confining the discussion to them. We should be glad to hear from anybody who will take part in the discussions.

DR. ROCHESTER: If I might be permitted to take the floor for a minute to talk a little about the X-ray. I have been very greatly impressed by the value of the X-ray in the study of the changes; of course not so much with the heart cases as in the pulmonary cases, and as the writer took up pulmonary cases quite extensively I don't think there is any positive necessity for taking them up. In the study of tubercular cases we find a great deal of value in the X-ray. We have been able by means of the X-ray, in connection with our physical examination, to place lesions very closely, locate them very closely indeed, and very incipient ones, too, with the aid of the X-ray. I do not think the X-ray can take the place of physical examination by any means, but it helps a great deal and is of great assistance to us. I would like to relate one case, not tubercular, in which the X-ray pointed out to us a very valuable finding. I was called to see a patient in consultation with this history: of cough, which had lasted for several months, and expectoration of a large amount of material at one time, and then subsequently, the old history of cavity emptying and filling up again, and of course there was a very bad smell with this expectoration, which gave us the idea of bronciectasis, and he had all the symptoms. By physical examination, the only thing we could demonstrate was a decided dullness in the right ventricular region between the second and fourth ribs, and we could not get any of the ausculatory signs in the cavity at any time, even when the cavity was empty or when he had raised a large amount of sputum; we could simply get dullness; but under the circumstances we took him up and fluoroscoped him, and I never saw a more beautiful exhibition of a cavity with fluid in it than that. The cavity was entirely filled, and as he breathed we could see the fluid move in it.

It was perfectly beautiful, it was more beautiful than the ordinary hydro-pneumo-thorax, because we expect to see that move, but this was very beautiful indeed. This was not a tubercular case, it was a fibrated lung with this bronci-ectasis. I took him to the hospital to drain, and Dr. Russell, who was acting as Dr. Sutter's assistant then, thought that he would use the artificial pneumo-thorax instead of draining this case, and we used the artificial pneumo-thorax, and only once did we produce the nitrogen. The result was perfectly beautiful, and I examined the man three days ago and he apparently had a perfectly normal chest, he was not spitting up anything, he was not coughing at all, he gained a great deal in weight, and I told him to come down—it was only yesterday that I examined him—and I told him to come down to the office so that we could fluoroscope him again, and I am sorry I am not able to give you the result of that in that case. It was quite remarkable to me. We owe the placing of that entirely to the X-ray.

The PRESIDENT: I will ask the writers of the papers now to reply to the discussion. Dr. Ross, will you begin?

DR. ROSS: In behalf of Dr. Loudon and myself, I cannot resist the temptation to again express our very heartfelt thanks for your kindly and appreciative welcome, especially because though we are quite near you, yet we are outside.

The PRESIDENT: Dr. Ross, we shall be very glad to see you again.

DR. PALMER: I have very little to say, except to thank those who discussed the paper. Dr. Doust made one statement, which I am not quite clear in my mind, what he meant by latent cases. Will you please explain, Dr. Doust, what you meant by a latent case?

DR. DOUST: I meant those cases in which there is some tubercular infection and the individual may never have known of it, but which has become healed.

DR. PALMER: I hardly agree with Dr. Doust on that, because you must take into consideration that tuberculosis is a case of tissue formation, fibrosis; that that forms an inflammatory condition which shows density, and therefore it is bound to show on the plate, not always on the flat single plate but on the very fine stereoscopic plate. We have been very negligent in these early cases of tuberculosis in not making stereoscopic Roentgengrams. They stand out beautifully. If you ever have an opportunity to look over Dunham's work and Wesley's work, of the University of Minnesota, do so. They have done wonderful work, and they have checked their work in this way and they correspond absolutely. Separate investigations. These little ramifications, as shown on the stereoscopic plate, stand out as little leaves or buds on a tree, and you can see little infiltrations. In the old cases they are more dense, they give you a denser shadow, and it seems to me it is still more easy. It is the early incipient cases which follow along the bronchus as it divides here that are so hard to make out, and I must confess that I have got a lot to learn yet in these early cases, and the more we do of them the more accurate we will become in making a diagnosis of early tuberculosis.

The PRESIDENT: I am very sorry to have to announce to the members that one of the main participants in this meeting, and from whom I know we all expected a great deal of interest to us, a man well known around this part of the world, is sick and not able to come. I refer to Dr. Elsner of Syracuse. It was with great regret that I received a letter from him a day or two ago saying that the doctors told him to go to bed, and that he did not dare to disobey. He wished me to present to the Society his best wishes for a successful meeting, and to regret, as I know we all regret, his inability to participate in this meeting. His subject I know would have been of considerable interest to you, and I wrote him in the hope that he could send his paper up, but he sent word that his paper was going to be largely written in his mind, and it was therefore not possible to have somebody represent him; so we will pass on now to No. 8, The Treatment of Cardio-Vascular Disease, by Dr. Stockton, of Buffalo.

DR. CHARLES G. STOCKTON, of Buffalo, presented a paper entitled: "Treatment of Cardio-Vascular Disease." (Published in December issue).

DR. LARKIN: Mr. President and members of the Society: I think we are to be congratulated upon this very excellent paper of Dr. Stockton's, but I feel that we are left a great deal in the same position that we have been in in regard to treatment of cardio-vascular diseases in the past, that it is largely a matter of prevention. The prophylactic treatment is the treatment par excellence, and after the ravages of the disease have once set in we have the changes in the circulatory apparatus and we are almost powerless to overcome these changes. Of course we can treat the patient symptomatically and relieve a great many of the passing and distressing symptoms which they have, but I believe that in these cases we have to search for the source of the infection, for many times it is due to an infection. Syphilis I think causes a large number of our cases of cardio-vascular disease. Infections in the mouth or in the tonsils or coming from the intestinal tract are also a large source of the cause of so-called arteriosclerosis. What effect the ductless glands may have on this condition I think is still an open and debated question. I am in hopes that some day some one is going to discover that the ductless glands do play more or less of an important part in this and probably help us out in our medication.

DR. ALLEN JONES: Mr. President, there are a few points I think well worth discussion in this admirable and comprehensive paper which Dr. Stockton has read. As Dr. Larkin says, prevention of course is of extreme importance, but Dr. Stockton's paper was on the treatment of cardio-vascular disease, and of course he could not take up that side. I was glad to hear Dr. Larkin mention the importance of the tonsils as ports of entry of infections causing renal disease. I think they are extremely important as a common cause of renal disease. Dr. Stockton mentioned three types of cardio-vascular renal disease, and his dissertation is very comprehensive. The first type of valvular disease illustrates the changes which take place in the kidneys and in the arteries, followed by a remarkable compensatory development on the part of the heart. Two cases I have in mind which illustrate that very particularly. One woman about twelve years ago had oedema, cardiac insufficiency. She had the congested kidneys at that time. She finally developed a high blood pressure and a very marked hypertrophy, got entirely rid of her oedema, and lived without the oedema, as I say, for twelve years, and lived for the latter years of her life with a high blood pressure. Those are quite remarkable cases. Another case I have in mind has done the same thing. It is remarkable that a heart disability and insufficiency could do this thing. What is there in the case which enables the heart to do that which we have very great difficulty in causing the heart to do by remedies in the other cases? The ductless glands may play a large part here. Dr Larkin has mentioned them in helping along to do just that thing which will prolong the life of the individual and give rise to the cardio-vascular hypertrophy which enables her to live. The Doctor mentioned in these classifications a matter of oedema and spoke of the digitalis and other remedies for the control of the situation and the oedema. I have found in those cases that the Carrel diet is valuable. Indeed, it was in the cardiac insufficiency cases Carrel first fell into the habit of using this diet. It was largely used, I think, in Hamburg, in one of the large hospitals in Hamburg, not by Carrel, but later by one of the physicians there, a well known physician. The Carrel diet then in oedema of this sort is extremely valuable, and another remedy which I have found useful when digitalis fails as a diuretic is theocin. In two cases, stopping digitalis and giving a fair amount of liquid and using theocin in five grain doses for two or three days has resulted in the passage of an enormous quantity of urine and a very rapid subsidence in the oedema. In the second series of cases Dr. Stockton mentioned an entirely distinct type of renal disease occurring in young people, in whom the blood pressure depends upon the renal disease and not upon an associated arterio-sclerosis, and this case the Doctor mentioned in the paper had a blood pressure of 240, and coming into the hospital for final visit. She had been travelling in the west, in no condition to travel, had gone out to the coast, and was on her return east, to her home in the east; thoroughly tired out, thoroughly exhausted, and the picture of that condition. Her blood pressure dropped from 240 to 130 in 24 hours. She temporarily, one day, felt better, but went into coma two days following and died. Coincident with the drop in the blood pressure there was amenorrhoea, a very scanty urine; so that merely illustrates the importance of the high blood pressure in maintaining life in these individuals, showing the primary influence of the kidney and the distinct nephritic side of these cases. The third class of cases, of chronic interstitial nephritis, the type that Dr. Stockton has so well described, I think an old observation made by many practitioners is worth remembering, and that is that in those cases, in the early stages of those cases, if we happen to observe them carefully and systematically, a chronic low specific gravity of the urine is of great importance, and it is in those cases particularly that I have had occasion to notice a galloping rythm. In no other cases have I seen galloping rythm so often or quite so typical as in that case of chronic interstitial nephritis. My experience has taught me to regard galloping rythm as a very grave prognostic sign. The patient may temporarily improve, but the condition must be regarded as most serious. The Doctor mentioned pheno-sulpho-pthalein. and there is just one point regarding that product which I wish to mention, and that is, that while the test is of great value as ordinarily used in a routine way in cases, yet when the arm is tremendously fat and the product is injected by yourself or by a nurse into the fat, and the first two hours urine is gathered, there may be a false reading. The whole of that product is not absorbed and the test proofs are extremely low; whereas, if three hours more are allowed to elapse a different result is obtained. A long needle should be used to go through the fat and reach the muscles, so as to make an intra-muscular injection. It would be desirable to use the injection intravenously, I mean to people with enormous adipose.

DR. STOCKTON: Mr. President, I do not want to go away with the feeling that I have been leaning too much on these newer studies in acidosis, but they have been to me of intense interest during the past year, and I at first was very much confused over their interpretation. The work of Palmer and Henderson of Harvard, and others in Boston and New Haven, whose publications you have doubtless read in the archives of Internal Medicine, most of them have been published, and in the Journal of Physiology,—you doubtless have been, as I was, interested to see what these things might indicate, and how they might be applied to clinical medicine. What I have been attempting to do this year, and this paper was intended to show in part that these pathological studies have a very definite clinical valuation, and I am sure that I know more about the progress of the case and understand certain otherwise mysterious things in a case by having the advantage of this work that these physiologists have so recently been doing, and those of you who have not given the matter attention, I beg that you will not turn the subject aside until you have looked into it and had it thoroughly tried by a competent physiologist chemist. There is no doubt at all that there is a big background in this subject of the hydrogen iron concentration, its bearing on acidosis, its relation to metabolism, and relation to heart and kidney functions. That is the one thing on which I want to lay emphasis before I sit down, because you might think I was getting rather too learned on the subject of hydrogen iron concentration. I know mighty little about it, I am trying to learn something about it, and I have through the help of those who know more been able to get something out of it. That is about all. Thank you very much.

In the absence of Dr. A. J. Colton, of Buffalo, the Secretary read his paper entitled: "Tuberculosis as we should view it."

THE PRESIDENT: This paper of Dr. Colton's is open for discussion. Has anybody any remarks to make on this paper? If not, this marks the conclusion of our meeting. I want to thank the various members who have taken part in this meeting for their co-operation in the success of today, and I now declare the meeting adjourned.

---

**The Elmira Academy of Medicine** held a regular meeting Feb. 2, Dr. C. F. Abbott of Elmira presenting Two Bladder Cases, and Dr. O. J. Bowman a paper on Tobacco Heart.

---

**The Rochester Academy of Medicine,** Section II, met Feb. 9. Dr. Wm. W. Percy gave a paper on Diagnosis and Surgical Treatment of Affections of the Pancreas and Spleen; Dr. Charles Reitz on Diagnosis and Surgical Treatment of Diseases of the Rectum, with Special Reference to Early Diagnosis of Cancer.

---

**The Buffalo Academy of Medicine** has held the following meetings since our last report: Jan. 26, Pathology, Latent Syphilis, with lantern slides, Dr. Aldred S. Warthin of Ann Arbor.

Feb. 2, Stated Meeting under auspices of Section of Surgery, Indispensability of X-ray in Treatment of Fractures, with lantern slides, Dr. C. R. Borzilleri; Inhalation Analgesia and its Application to General Surgery, Dr. John H. Evans.

Feb. 9, Medicine, Syphilis and Epilepsy, Dr. Wm. T. Shanahan. with Pathologic Reports by Dr. J. F. Munson, Craig Colony for Epileptics, Sonyea. Moving Pictures of Epileptic Seizures, Dr. A. L. Shaw, same place.

Feb. 16, Obstetrics and Gynaecology, Twilight Sleep, Dr. Wm. T. Getman; Nitrous Oxid-Oxygen, Dr. N. Sproat Heaney, Chicago.

Feb. 23, Pathology, The Public Health Laboratory in its Relations to the Community, Dr. Rogen G. Perkins, Cleveland.

---

**The Gross Medical Club** held their annual meeting at the Hotel Touraine, Friday, February 18; Dr. J. Henry Dowd, president, presided; Mrs. C. J. Dwyer acted as hostess. An interesting paper, the Treatment of Rheumatism by Phylacogens, was read by Dr. H. C. Lapp of Tonawanda. The paper was discussed by practically every member present, the consensus of opinion being "There is no doubt of their efficiency in true rheumatism, but the first care should be used in the diagnosis; be sure you are treating rheumatism and not nerve involvement, as neuritis, neuralgia, etc."

Many interesting cases were reported, cases out of the ordinary run of every day practice.

Dr. D. C. McKinney,—A child of four months, well nourished, but every time she was fed, the bowels moved at once, accompanied by straining and pain. Examination revealed a horse shoe fistula, a rare condition at this age.

Dr. Phelps, East Aurora—A girl twelve years old, recovered, apparently, from chicken pox. About a week after was seized with chills and high fever, lobar pneumonia. Every day, from three to five p. m., temperature registered 105 to 105 4-10 F. It would then drop to almost normal. Careful examination revealed no tubercle bacilli. Fever continued for seven days and gradually dropped to normal; there was a perfect recovery.

Dr. Wilcox, Tonawanda—Patient developed tetanus, following a slight wound of the hand. 20,000 units tetanic serum was given every six hours for four or five days, or until spasm was released. It was then reduced but almost immediately returned, and the above dose was repeated every six hours until entire absence of symptoms. The patient made a good recovery.

Dr. Baker, Williamsville—An epidemic of grippe, occurring in an old folks' home, all patients from 65 to 80 years of age; 15 died. One peculiar phase of this epidemic was, practically all these patients, after several days of appropriate treatment, would be seemingly greatly improved, when suddenly they would die, apparently from cardiac failure.

Dr. J. Henry Dowd—Reported the following case because of the two quite rare accompaniments of a very common condition, still one which may arise at any time, giving you much alarm. Mrs. T. was asked to be seen in consultation for syphilis; a sample of urine was asked for before seeing her. This revealed no pathological condition. The phosphatic index was 20% minus, crystals normal in shape, but showing hysteria. The patient was found in bed, where she had been for two weeks, the only symptom she complained of being that she could not see, was blind. She was well nourished, had good appetite, bowels regular, no pain of any description. She could go to the bath room when necessary, but could not get up for meals or even to get a drink of water. Careful examination revealed no evidence of syphilis, in fact, she never had this disease. Fluid extract of valerian was ordered, and patient asked to be watched carefully, telling the family her condition was undoubtedly hysteria. In two weeks again, she was seen, being found in practically the same condition.

It was explained to the family that medicine would do her no good, and strenuous methods must be used. When she asked for water, she was to be told to get up and get it, and when meals were ready, they were to be announced as follows: "Dinner is now ready in the dining room, service is free." Under no condition was any food to be taken to her; twelve hours sufficed to bring her downstairs for her food, the blindness disappeared at the same time. The appearance of the alkaline crystals showed positively the condition before the patient was seen.

Dr. Geo. F. Cott—Consultation in case of difficult breathing revealed a large swelling on the larnyx. The trachea was opened and the patient slept well all night. She died very suddenly next day. There is no doubt that the condition was caused by bacillus La Grippe.

Case 2. Eleven years previous, this case was operated for abscess of the brain. This patient died suddenly a few days ago, and an autopsy revealed enlargement of the petrous portion and regeneration of the temporal bone, where it had been removed. Pus was found in the middly ear, but having no connection with the brain.

The election of officers resulted as follows:

W. Warren Britt, Tonawanda, President;

James E. King, Vice-president;

Chester C. Cott, Secretary.

A banquet followed at which Mme. Reister, the charming vocalist, rendered several selections. She was accompanied at the piano by her sister.

